# Diversity of bacteria populations associated with different thallus regions of the brown alga *Laminaria digitata*

**DOI:** 10.1371/journal.pone.0242675

**Published:** 2020-11-25

**Authors:** Maureen W. Ihua, Jamie A. FitzGerald, Freddy Guihéneuf, Stephen A. Jackson, Marcus J. Claesson, Dagmar B. Stengel, Alan D. W. Dobson

**Affiliations:** 1 School of Microbiology, University College Cork, Cork, Ireland; 2 APC Microbiome Institute, University College Cork, Cork, Ireland; 3 SAS inalve, Villefranche-sur-Mer, France; 4 Botany and Plant Science, School of Natural Sciences, Ryan Institute for Environmental, Marine and Energy Research, National University of Ireland Galway, Galway, Ireland; 5 Environmental Research Institute, University College Cork, Cork, Ireland; University of New South Wales, AUSTRALIA

## Abstract

Stipitate kelp species such as *Laminaria digitata* dominate most cold-water subtidal rocky shores and form underwater forests which are among the most productive coastal systems worldwide. *Laminaria* also sustains rich bacterial communities which offer a variety of biotechnological applications. However, to date, in-depth studies on the diversity and uniqueness of bacterial communities associated with this macroalgal species, their ecological role and their interactions with the alga are under-represented. To address this, the epibacterial populations associated with different thallus regions (holdfast, stipe, meristem, blade) of this brown seaweed were investigated using high-throughput Illumina sequencing of the 16S rRNA genes. The results show that epibacterial communities of the brown seaweed are significantly different and specific to the thallus region, with the shared bacterial population comprising of only 1.1% of the total amplicon sequence variants. The diverse holdfast and blade tissues formed distinct clusters while the meristem and stipe regions are more closely related. The data obtained further supports the hypothesis that macroalgal bacterial communities are shaped by morphological niches and display specificity.

## Introduction

Marine macroalgae (seaweeds) represent a unique source of high value hydrocolloids, such as agar, carrageenan and alginate, which have several biotechnological applications [1–3]. The global alginate market alone was valued at USD624.0 million in 2016 and is estimated to be worth USD923.8 million by 2025 [4, 5]. Overall, hydrocolloids obtained from seaweeds have a commercial value of up to US$1 billion which is also projected to further increase [6]. Extracts from seaweeds possess various pharmaceutical properties such as antiviral [7], anti-inflammatory [8] and anticoagulant activities [9], together with being demonstrated to alleviate abiotic stress in plants [10]. Laminarin derived from *Laminaria digitata* has for example, been demonstrated to reduce the infection of *Plasmopara viticola*, a disease-causing fungus; on grapevine by 75% by eliciting several defence mechanisms such as optimizing calcium influx, alkalinization of the extracellular medium and activation of defence-related genes [11].

Seaweeds are also desirable marine species for use as feedstock for ethanol fermentation, as well as anaerobic digestion for methane production due to their rich carbohydrate content [12]. *Laminaria digitata* which contains up to 57% glucose, has in particular been identified as one of the most promising brown seaweed biomass sources for biomethane production [13]. The lack of lignocellulose and the possibility of enzymatic saccharification at lower temperatures has also positioned marine macroalgae as a potentially better and more environmentally friendly alternative to the use of land plants for ethanol production [14]. Marine macroalgae have thus gained increased attention in recent times, as evidenced by an increase in the harvesting and cultivation of seaweeds of over 9 million tonnes between 2001 and 2010, which resulted in more than US$ 2,375M increase in revenue [15]. This increased seaweed farming has had the additional beneficial effect of lowering overfishing rates, and also improving the socio-economic lifestyle of communities in coastal regions [16].

Owing to the carbon rich nature of the algal cell walls and the physicochemical properties of seaweed surfaces, they provide a suitable substratum for colonization by epibionts and act as a shelter for many invertebrate species [17–19]. These seaweed-attached bacteria can exert essential functions relating to the positive health and development of their algal host [19, 20]. Metabolites such as thallusin from epiphytic marine bacteria for example induce differentiation and germination in *Monostroma oxyspermum* and other green macroalgae [21]. However, other opportunistic bacteria which may elicit negative interactions, such as bleaching disease in the red alga, *Delisea pulchra*, also exist and are increasingly being reported [22].

The structure of macroalgae-associated bacterial populations may be influenced by geographical location, host algal species composition, as well as functional differences between morphologically distinct parts of the alga [23]. Host species, tissue age, biogeography together with nutrient levels have been shown to contribute to the bacterial communities in *Caulerpa prolifera*, *Caulerpa cylindraecea*, *Nereocystis luetkeana* and *Macrocystis pyrifera* [23, 24]. On the other hand, microbial communities found on the meristem and stipe regions of *Laminaria saccharina* (*Saccharina latissima*) were found to be more closely related, regardless of geographical region or seasonal influences, while the epibacterial populations found on the blade and holdfast of the same alga were considerably different [25]. Previous studies on *L*. *digitata* also report that while the bacterial communities associated with the blade region of this brown alga are diverse, they are functionally different from the microbial communities present on the metabolically inactive aged peripheral tissues of the macroalga [26, 27].

This variation observed in the microbial populations associated with different morphological parts of macroalgae may be due at least in part to the non-vascular architecture of seaweeds. Honkanen and Jormalainen [28] suggest that different parts of most seaweeds exhibit independence with respect to the absorption of nutrients and the production of photosynthates, and lack vascular connections for efficient resource translocation. Transcriptome analysis of *Caulerpa lentillifera* [29] further reveal a differential gene expression in different segments of the green alga, each with distinct functions. It is thus logical to suggest that consequently, the bacterial communities associated with each morphological segment may differ.

Bacterial communities associated with *L*. *digitata* are yet to be fully described, with little currently known regarding the microbiome composition of the different morphological regions of the brown alga. In this study, the surface-attached microbial communities associated with four different regions (holdfast, stipe, meristem, and blade) of *Laminaria digitata* were characterized. Each region of the algal thallus was sampled over a 10-month period (April 2016, July 2016, November 2016, and January 2017) and investigated using high throughput Illumina MiSeq sequencing. Taxonomic analysis demonstrates that bacterial populations associated with *L*. *digitata* display significant morphological variation, with the holdfast being identified as the most diverse region, whereas the meristem region was found to be the least diverse. Certain bacterial amplicon sequence variants (ASVs) also showed preferential relative abundance to specific regions of the algal body. Overall, *L*. *digitata*-associated bacterial populations demonstrate high specificity, with only 1.1% of amplicon sequence variants being shared amongst the four thallus regions.

## Materials and methods

### Sampling

**Fig 1:**
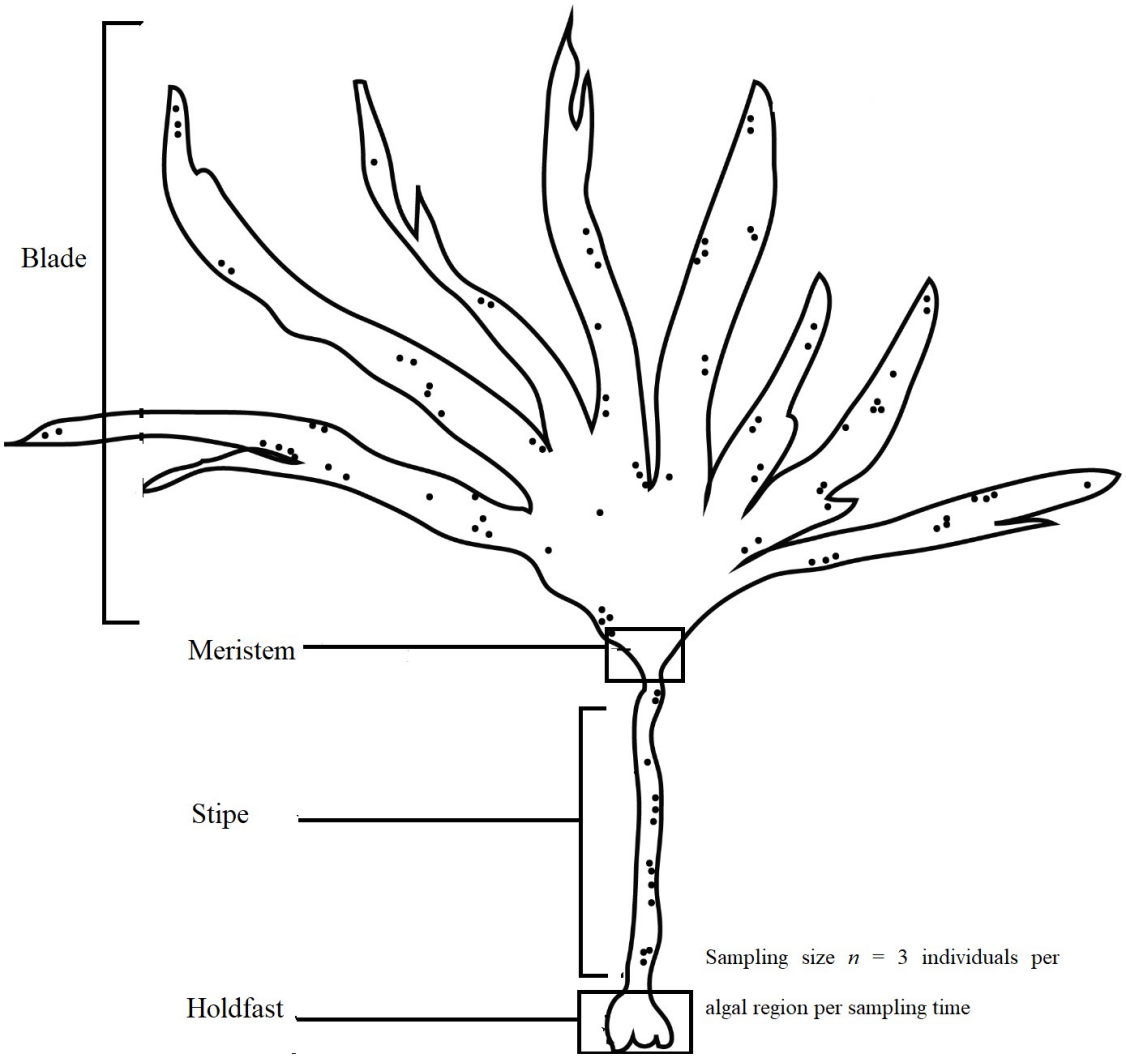
Four morphological parts of a *Laminaria digitata* thallus.

*Laminaria digitata* was obtained from Finavarra in Co. Clare, Ireland at 53° 08’ 59” North, 9° 08’ 09” West in April 2016, July 2016, November 2016, and January 2017. At each sampling time, three individual algal thalli were collected, each sectioned into four different regions (holdfast, stipe, meristem, and blade as shown in Fig 1; *n* = 3 individuals per algal region per sampling time), vigorously rinsed in sterile water to remove loosely attached particles and transferred into sterile air-tight plastic bags. All samples were kept on dry ice at the sampling location and subsequently stored at either -20 °C or -80 °C. No permits were required for sample collection as the sampling location is open for public access and use, and the alga was harvested in small quantities. The sample metadata is represented in S1 Table.

### DNA extraction

Metagenomic DNA was extracted from approximately 0.5 g (wet weight) of algal sample (*n* = 3 individuals per algal region per sampling time) which was randomly selected and crushed into a fine powder under liquid nitrogen using a sterile mortar and pestle. The ground powder was suspended in 500 ml of CTAB lysis buffer (2% *w/v* CTAB, 2% *w/v* polyvinylpyrrolidone, 1.4 M NaCl, 20 mM EDTA pH 8.0, 100 mM Tris HCl pH 8.0), bead-beaten for 60 seconds with sterile beads and incubated at 65 °C for 60 minutes, with occasional mixing. Equal volume of phenol chloroform isoamyl alcohol was added, and the mixture was centrifuged for 30 minutes at 4,300 x *g*. The aqueous phase was collected into a separate Eppendorf tube, and the DNA was precipitated with 0.7 volume of ice cold isopropanol and 0.1 volume of sodium acetate (3 M, pH 5.2) and centrifuged (at 20 °C) for 30 minutes at 4,300 x *g*. The pellet was washed twice in 70% *v/v* ice cold ethanol, air dried and re-suspended in TE buffer. DNA was visualized using agarose gel electrophoresis and quantified using Nanodrop spectrophotometer (Thermo Fisher Scientific, Delaware, USA).

### 16S rRNA gene amplification and MiSeq sequencing

Metagenomic DNA extracted from each of the three individuals for each algal region at each sampling time (*n* = 3 individuals per algal region per sampling time) were pooled together in equal volumes for PCR amplification of the V3-V4 16S rRNA gene region which was performed using forward (5’- *TCGTCGGCAGCGTCAGATGTGTATAAGAGACAG*CCTACGGGNGGCWGCAG -3’) and reverse (5’- *GTCTCGTGGGCTCGGAGATGTGTATAAGAGACAG*GACTACHVGGGTATCTAATCC -3’) primers [30] as previously described [31]. This set of primers which contain Illumina adapter overhang sequences (written in italic text) have previously been identified as being efficient in representing good levels of bacterial diversity and have been used in a wide range of environmental studies [30, 32, 33]. PCR amplification was performed (using 1–10 ng DNA) under the following conditions: 98 °C for 30 s, followed by 30 cycles of denaturation (98 °C for 10 s), primer annealing (57 °C for 30 s), primer extension (72 °C for 30 s), and 72 °C for 5 min. PCR amplicons were purified using Agencourt AMPure XP beads (Beckman Coulter) according to the manufacturer’s instructions and a subsequent reduced-cycle (8 cycles) reaction was performed to further attach unique dual eight-base Nextera XT multiplexing indexes and sequencing adapters under the following cycling conditions: 98 °C for 30 s, followed by 8 cycles of denaturation (98 °C for 10 s), primer annealing (57 °C for 30 s), primer extension (72 °C for 30 s), and 72 °C for 5 min. Index PCR products were purified using Agencourt AMPure XP beads (Beckman Coulter; Fisher Scientific, Dublin, Ireland) according to the manufacturer’s instructions. For each pooled metagenomic DNA (pool of 3 individuals per algal region per sampling time), PCR reactions were performed three times. Equal volumes of respective triplicate amplicons were then pooled together and sequenced using Illumina Miseq 300bp paired-end sequencing by Macrogen (Seoul, Korea).

### Sequence data processing and statistical analyses

Raw sequence data obtained were de-multiplexed according to their unique barcodes and processed using Trimmomatic (version 0.38) [34] to remove end positions with a quality score below 25. The R package DADA2 [35] was then used to exclude primer sequences, filter and de-noise sequences, de-replicate unique amplicon sequence variants (ASVs, similar to 100%-identity operational taxonomic units), and remove chimeric sequences. R package DADA2 default settings were employed for these analysis, with the exception of maximum error (maxEE = c(3,4)). Taxonomic identities of ASVs were assigned using the R package DECIPHER [36] and the SILVA 132 database release [37]. Chloroplast sequences were identified were and removed from downstream analyses. Table 1 shows the number of raw read-pairs and quality filtered sequences, together with the normalized sequencing depth of each sample. Ecological metrics were calculated using the R package vegan [38], and read counts were normalized using the R package GMPR [39]. Differential testing between conditions was carried out using ADONIS2 and ANOSIM (R package vegan) [38], as well as a Kruskal-Wallis test for differences between groups with post-hoc Dunn test (R package FSA) [40]. Cluster analysis of samples by Bray-Curtis distance was carried out via Ward-linkage (*hclust*, method = "ward.D") [41], while abundant ASVs (at least 300 reads in at least 15% of samples) were hierarchically clustered using the complement of Spearman correlation between the relative abundances of each ASV.

**Table 1:** Number of raw read-pair counts, quality-filtered and chloroplast removed sequences, normalizing correction applied, and final normalized read abundances of bacterial communities associated with the holdfast (HF), stipe (SP), meristem (MST) and blade (BD) of *L*. *digitata*.

Sample ID	Number of raw sequences	Number of final sequences (quality filtered, and chloroplast removed)	Normalization size-factor (GMPR)	Normalized sequencing depth
BD1	695505	34334	1.5328674	52632
MST1	584181	62629	1.5893738	99540
SP1	665880	123514	2.0303603	250786
HF1	736848	120233	1.0561296	126965
BD2	784215	31330	0.8036972	25201
MST2	596318	15277	1.3692453	20923
SP2	705793	29591	1.9676569	58230
HF2	765337	46732	0.5761058	26927
BD3	709843	38899	1.4675438	57152
MST3	709310	15768	0.4573797	7214
SP3	726262	55180	0.6715527	37080
HF3	599843	154133	0.2586415	39860
BD4	661431	34348	1.2218654	41962
MST4	699441	46596	1.0434568	48624
SP4	637670	25971	2.048081	53203
HF4	657932	83347	0.5040371	41994

A correlation network between features (ASVs) was constructed using Spearman’s correlation coefficient and the R package igraph [42]. Relative abundances were compiled and presented in Graphpad [43], while community features were visualized and explored using the R packages ggplot2, phyloseq, complexheatmap, vegan, gplots, venneuler and reshape2 [38, 44–49]. The final ASV table (S2 Table) and taxonomy table (S3 Table) are provided in the Supplementary materials.

### Accession number

The 16S rRNA amplicon sequencing data was deposited in the European Nucleotide Archive (ENA) under the accession number ERX3500545—ERX3500549, ERX3500553-ERX3500560 and ERX3524768—ERX3524771.

## Results

### Diversity of *L*. *digitata* communities

**Fig 2:**
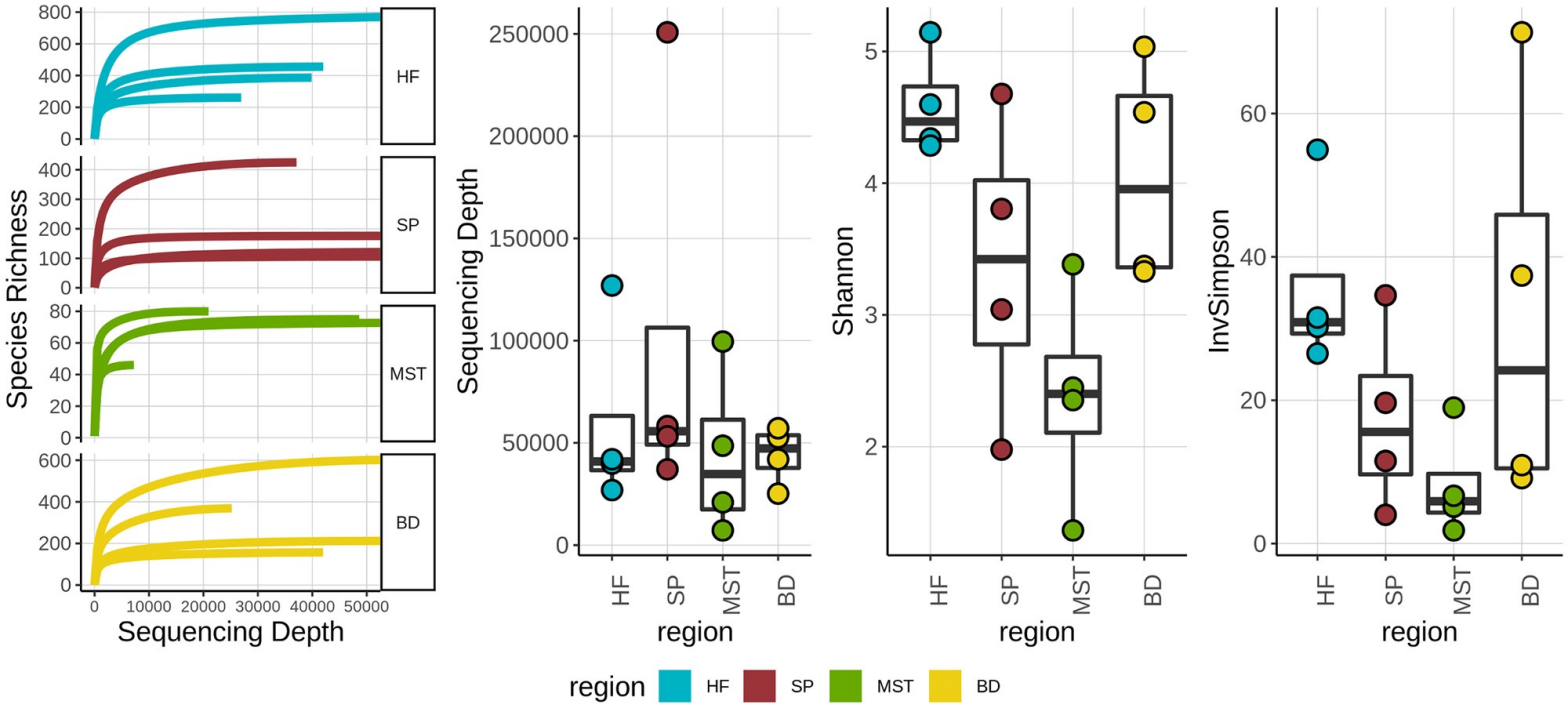
Species richness, sequencing depth and alpha diversity measures of the microbial communities associated with holdfast (HF), stipe (SP), meristem (MST) and blade (BD) of *L*. *digitata*. Boxplot; middle is median, outer margins are 25th & 75th percentiles; whiskers cover points within 1.5 interquartile ranges.

Epibacterial communities derived from the holdfast, blade, stipe and meristem regions of *L*. *digitata* were analyzed to determine if differences existed in their overall structure, diversity, and composition. The diversity of the bacterial communities was examined using Shannon and Inverse Simpson indices represented in Fig 2. Overall, the *L*. *digitata* holdfast region displayed the highest level of species richness and diversity, closely followed by the algal blade. The meristem was also found to be the least diverse, while stipe displayed medium levels of diversity (Fig 2).

**Fig 3:**
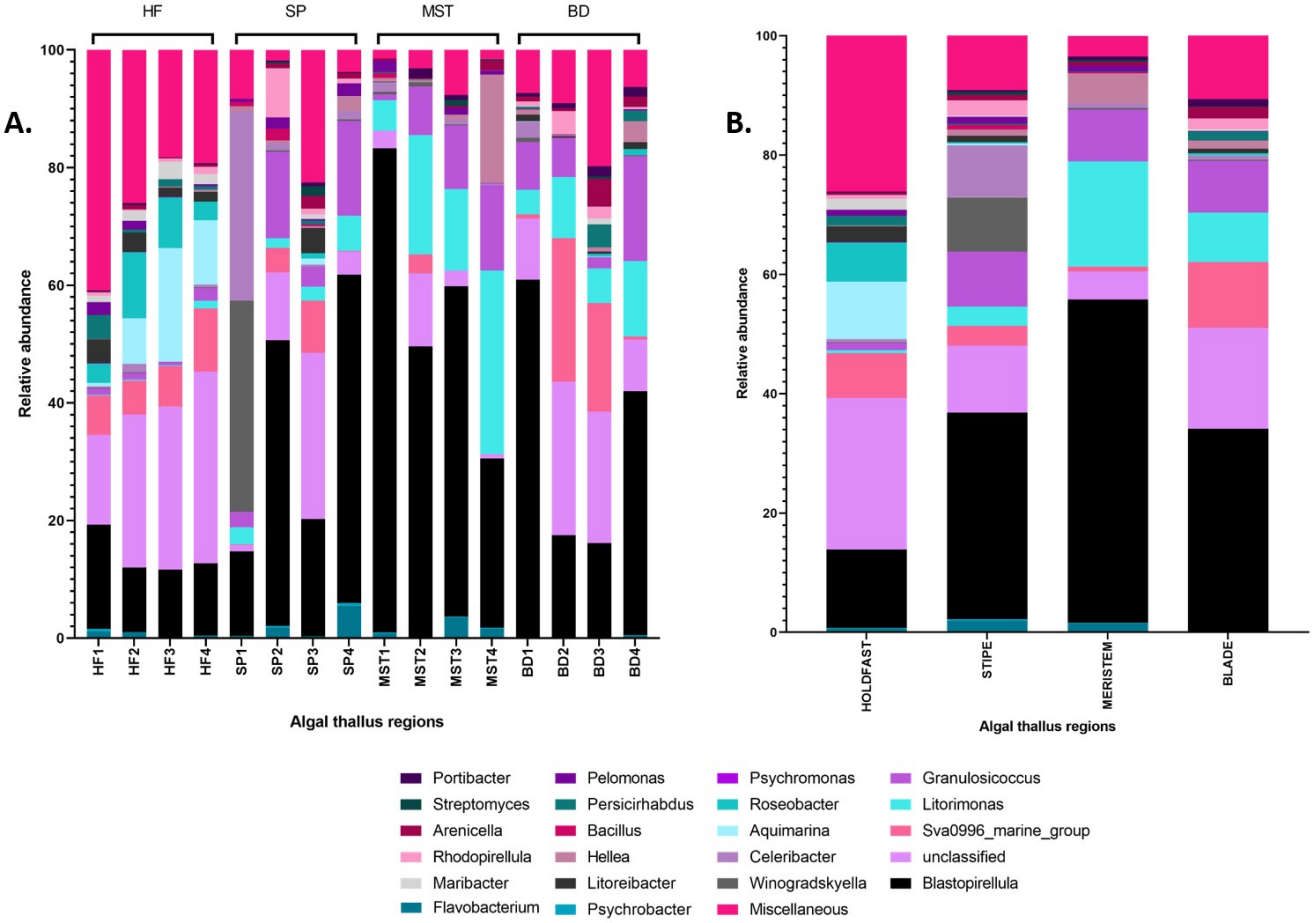
(A) Relative abundance of individual samples and (B) average relative abundance of corresponding samples at genus level of the holdfast (HF), stipe (SP), meristem (MST) and blade (BD) of *L*. *digitata*. Miscellaneous; bacterial phyla with less than 0.005 rank mean sample relative abundance.

The bacterial communities associated with *L*. *digitata* consist of bacteria which belong to the phyla *Proteobacteria* (40%), *Planctomycetes* (36%), *Bacteroidetes* (12%), *Actinobacteria* (8%), *Verrucomicrobia* (2%), *Cyanobacteria* (1%) and *Firmicutes* (<1%). At the genus level after classifying with the SILVA database, 15% of the bacterial ASVs remained unclassified while up to 21 genera, including *Aquamarina*, *Blastopirellula*, *Flavobacterium*, *Litorimonas* and *Hellea*, were identified (Fig 3). These unclassified ASVs are likely to represent groups of bacteria currently unavailable in the SILVA database (version 132) [37] which we employed in this study, or possibly novel bacterial species.

**Fig 4:**
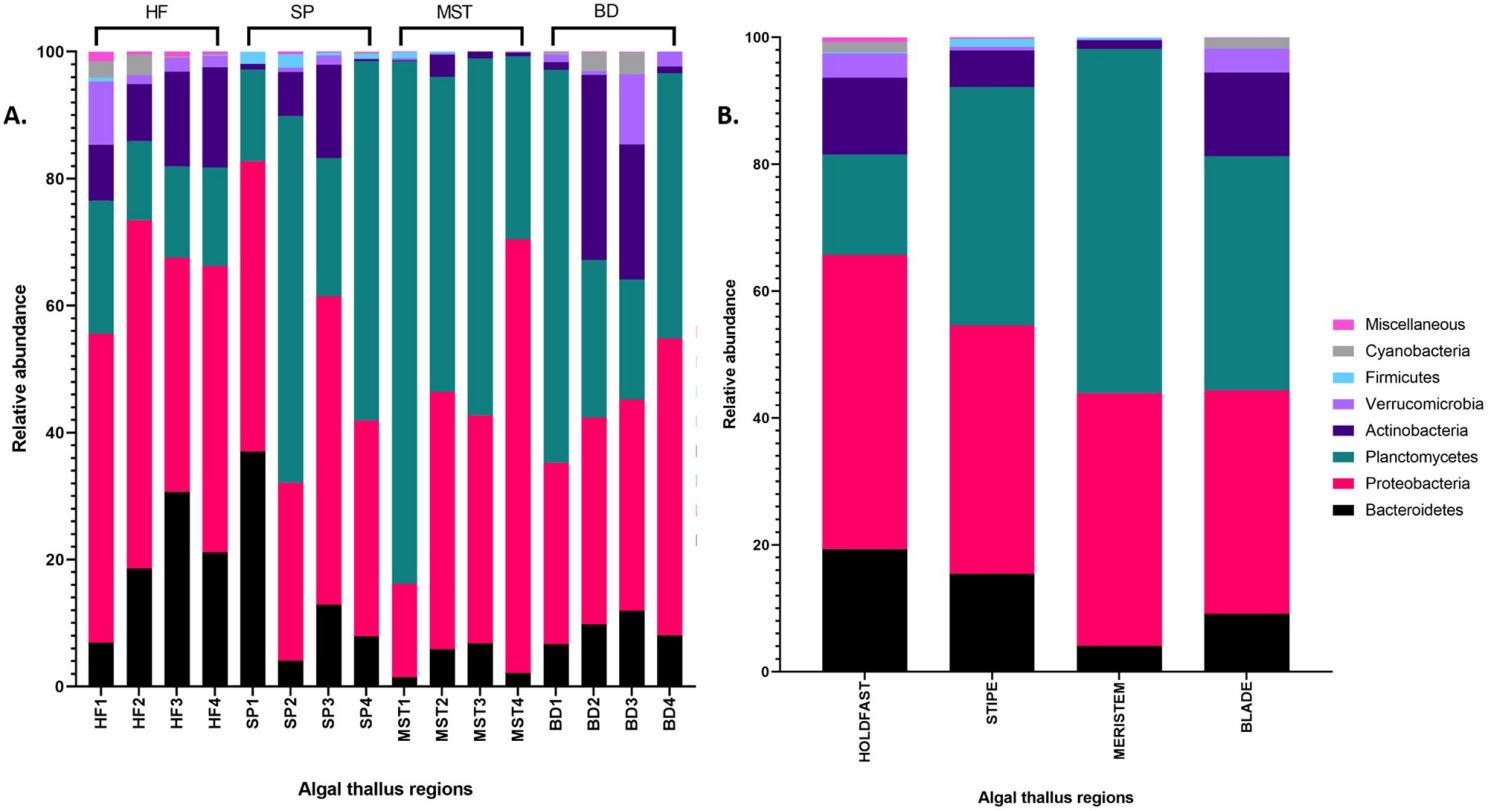
(A) Relative abundance of individual samples and (B) average relative abundance of corresponding samples at phylum level of the holdfast (HF), stipe (SP), meristem (MST) and blade (BD) of *L*. *digitata*. Miscellaneous; bacterial phyla with less than 0.005 rank mean sample relative abundance.

Distinct differences exist between the epibacterial microbial populations associated with the four different regions of the brown seaweed (Figs 3 and 4). The holdfast bacterial community was dominated by the phylum *Proteobacteria* (46%), followed by *Bacteroidetes* (19%), while *Cyanobacteria* (1.6%) and *Firmicutes* (0.2%) were the least observed. Diverse genera such as *Blastopirellula* (13%), *Aquamarina* (9.6%), SVA0996 marine group (7.5%), *Roseobacter* (6.6%), *Litoreibacter* (2.7%) and *Maribacter* (1.9%) were identified in the holdfast, but remarkably, up to 25% of ASVs remained unclassified at the genus level. This is the highest average relative abundance recorded for unclassified sequences amongst the four thallus regions, and could potentially represent novel bacterial groups within the holdfast community. The stipe region on the other hand was characterized by the presence of *Blastopirellula* (34.7%), *Granulosicoccus* (9.2%), *Winogradskyella* (9%), *Celeribacter* (8.8%), SVA0996 marine group (3.3%), *Rhodopirellula* (2.5%) and *Flavobacterium* (2%). Interestingly, *Blastopirellula* (54.2%) was identified as the dominant bacterial genus present in the meristem-related bacterial community, while notable levels of other genera such as *Litorimonas* (17.6%), *Granulosicoccus* (8.6%) and *Hellea* (5%) were also observed. The highest relative abundance of the genus SVA0996 marine group (11%) was observed in the blade region, whereas, members of the genera *Flavobacterium*, *Aquamarina* and *Pelomonas* were scarcely present, each at ≤ 0.1%.

### Comparative analysis of *L*. *digitata*-associated bacterial populations

**Fig 5:**
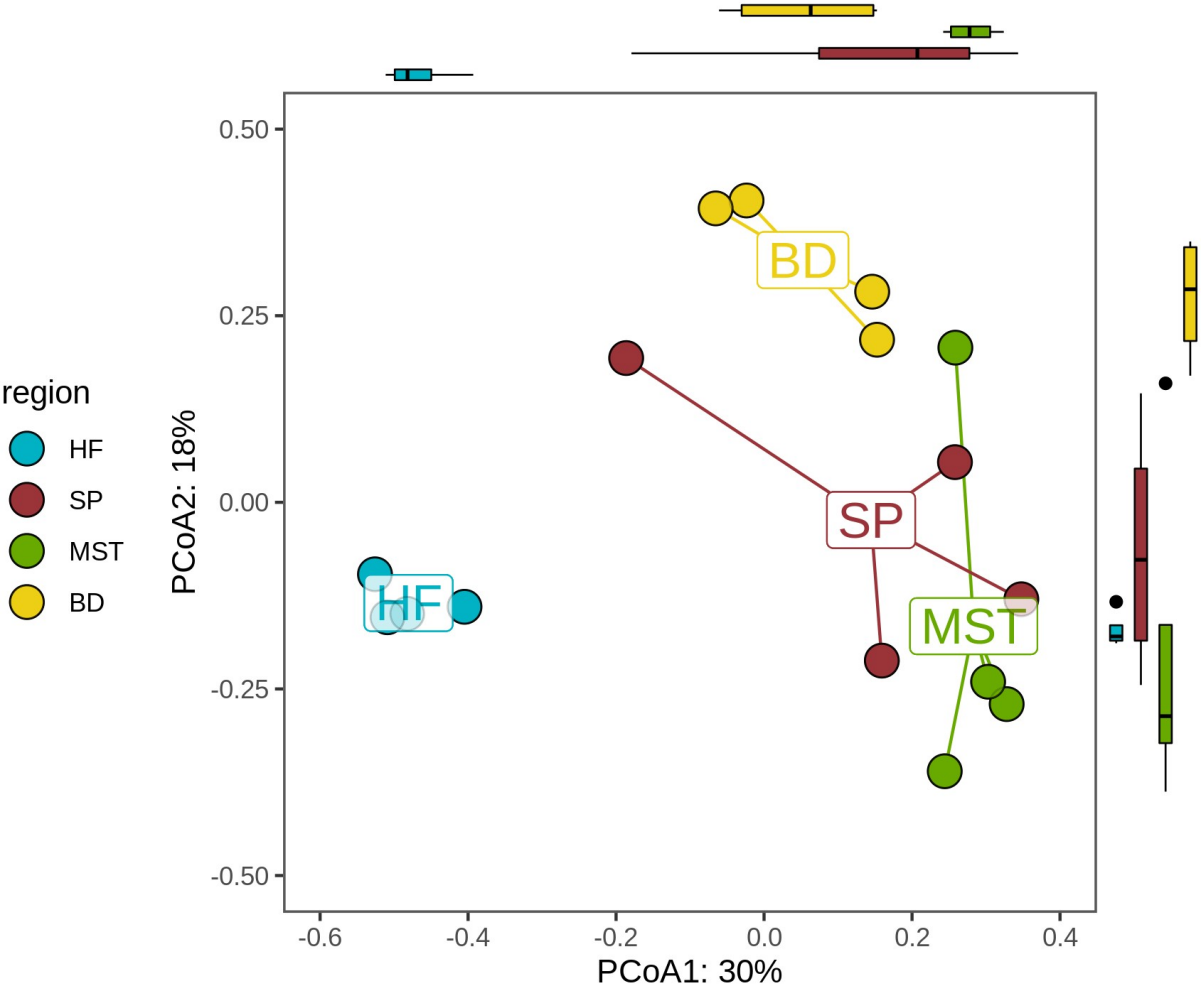
Principal coordinate analysis (PCoA) plot based on Bray-Curtis distance matrix of the epibacterial communities associated with the holdfast (HF), stipes (SP), meristem (MST) and blades (BD) of *L*. *digitata*.

Differences in community composition (beta diversity of Bray-Curtis dissimilarity values) are represented in the PCoA ordination plot (Fig 5). The margins show how well the samples are separated within the first two axes, which account for 48% of the variation observed, with a boxplot emphasizing the segregation of samples based on seaweed region (middle is median, outer margins are 25th & 75th percentiles; whiskers cover points within 1.5 interquartile ranges). Centroids represent the average value for all samples within each region category, while the distance between the positions of any two plots represent the extent of their dissimilarity in bacterial composition. The holdfast-related bacterial communities associate strongly, forming a distinct cluster which is separate and distantly located from the other bacterial populations (Fig 5). Similarly, the blade-related bacterial communities displayed strong similarities and formed separate clusters, while, the microbial populations from meristem and stipe are more closely related than they are to holdfast and blade related communities. A Kruskal-Wallis test (S4 Table; chi-squared = 11.404, df = 3, p value = 0.009729) of the variance contained in the first two axes of the PCoA plot revealed statistically significant differences between the epibacterial communities associated with the four different algal thallus regions. Furthermore, a post-hoc Dunn test identified holdfast versus stipe (S4 Table; Z score = -2.451, adjusted p value = 0.043) and holdfast versus meristem (S4 Table; Z score = -3.193, adjusted p value = 0.008) as the comparisons which explain the significant differences in associated epibacterial populations observed between the algal regions. The fact that holdfast is present in both significant pairs, while meristem versus stipe (S4 Table; Z score = 0.743, adjusted p value = 0.458) is not significantly different indicates the holdfast is the most significantly different region.

### Core and unique epiphytic bacterial communities on *L*. *digitata*

**Fig 6:**
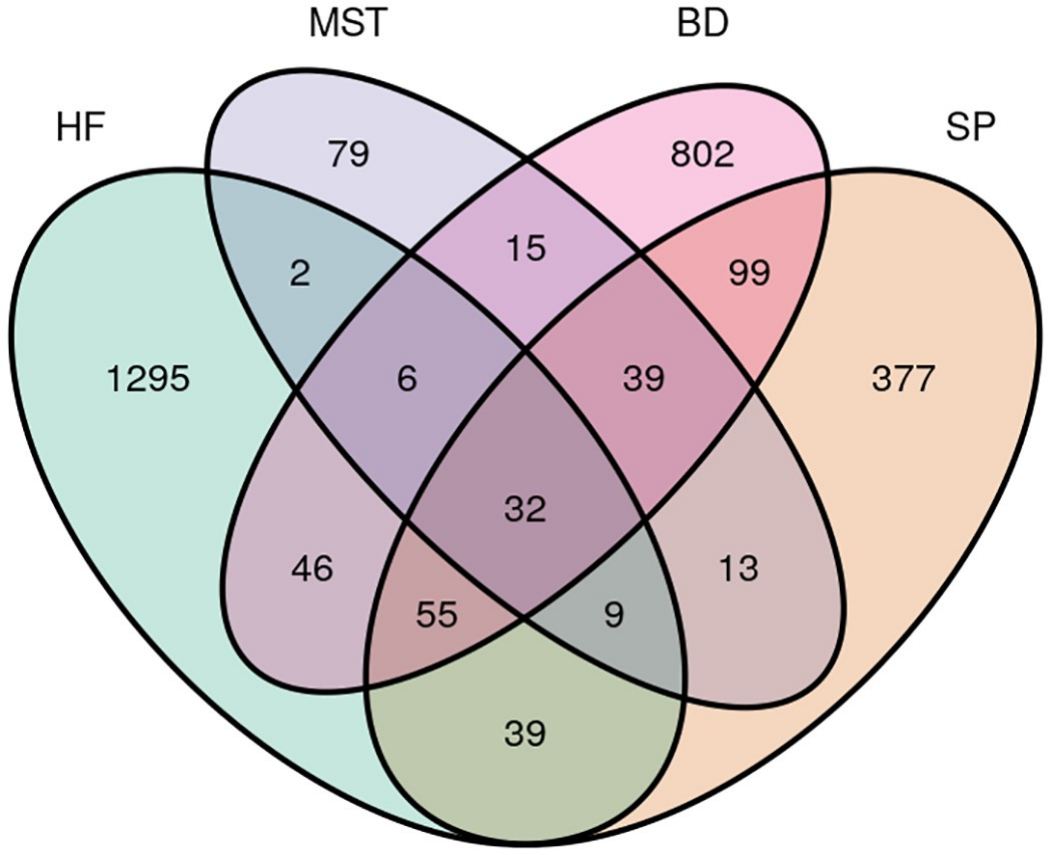
Venn diagram showing unique and overlapping amplicon sequence variants (ASVs) identified in *L*. *digitata* bacterial communities categorized region. Abbreviations; holdfast (HF), meristem (MST), blade (BD) and stipe (SP).

The epibacterial communities which are unique to each algal thallus region (Fig 6), as well as bacterial species shared across all samples were analyzed. Grouping through Venn diagrams was carried out on all normalized ASV abundances (as provided in Table 1) and as such, Venn groups were defined on a presence/absence basis across the study. The holdfast region displayed the highest number of unique ASVs (45%; 1295 ASVs), suggesting a microcosm suitable for the colonization of a specific subset of bacterial species. Only 2.7% (79 ASVs) on the other hand were unique to the meristem region, while blade and stipe harbored 802 and 377 unique ASVs, respectively. Overall, *L*. *digitata* surface-attached bacterial populations are specific, with only 1.1% ASVs (32 of 2,908 ASVs) being shared between the morphological regions. Most of these shared communities were further identified as belonging to the genus *Blastopirellula*.

**Fig 7:**
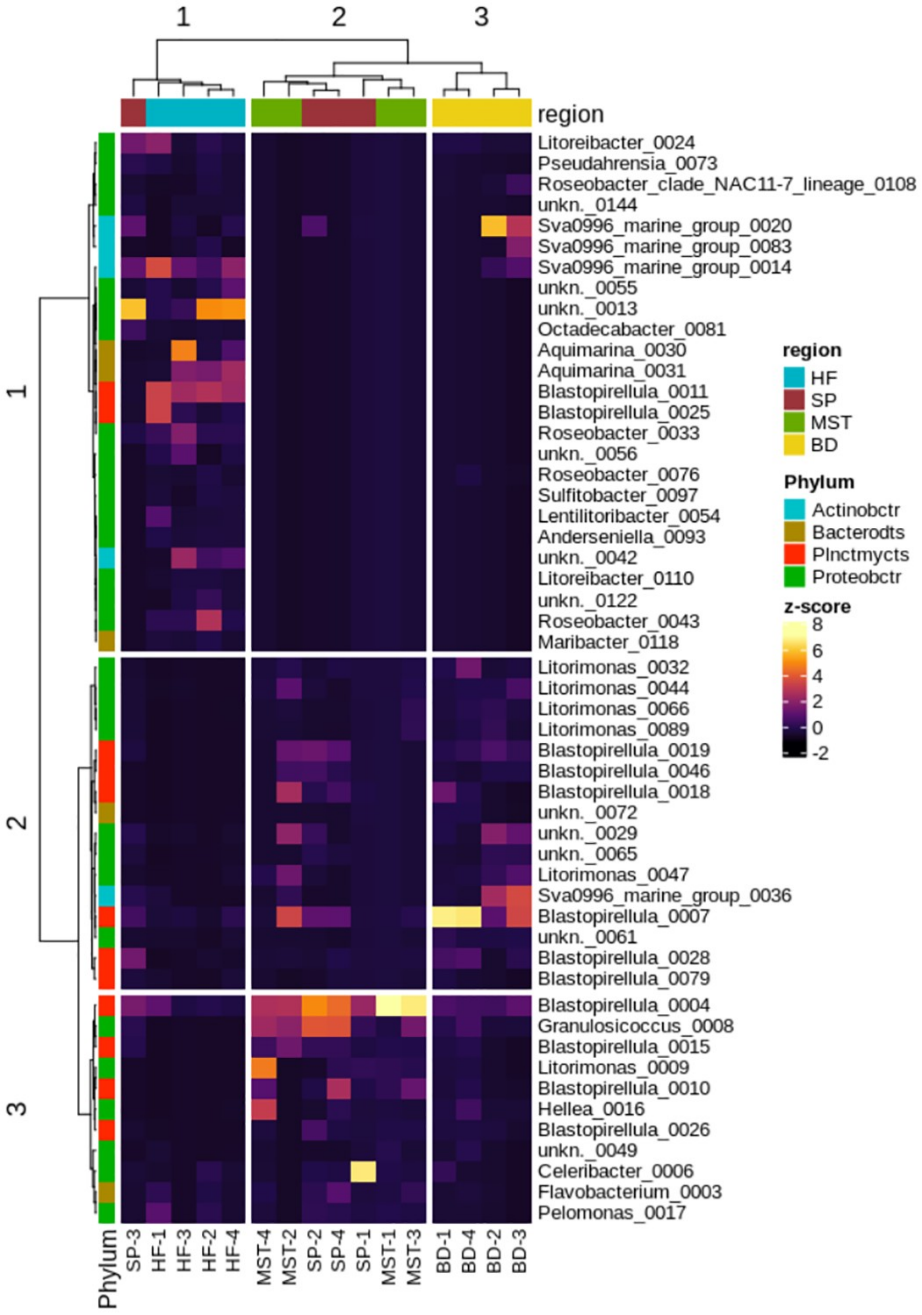
A heatmap of the 52 most abundant ASVs illustrating the broad differences in community composition between locations on *L*. *digitata*. Abundances are normalized to unit-variance (z-score transformation). Abbreviations: Proteobactr: *Proteobacteria*, Bacterodts: *Bacteroidetes*, Plnctmycts: *Planctomycetes*, Actinobctr: *Actinobacteria*, SprW: Spearman correlation with Ward-linkage, HF: holdfast, SP: stipe, MST: meristem, BD: blade.

Clustering of samples by community dissimilarity (Bray-Curtis) corresponded well with the source of the sample, with both blade and holdfast presenting distinct communities while meristem and stipe regions cluster together (Fig 7), possibly reflecting the similar ecological niches they represent to epiphytes. Multiple ASVs across the sampling regime represent some genera (*Blastopirellula*, *Litorimonas*, Sva0996 marine group). In particular, *Blastopirellula* variants are found across the seaweed but show preference for specific locations on the algal body: *Blastopirellula*_0004 is most abundant at the stipe and meristem, while *Blastopirellula_0007* is more abundant on the seaweed blade, and *Blastopirellula* 0011 is most abundant at the holdfast, suggesting niche adaptations within the genus.

## Discussion

Over a decade ago, Staufenberger and co-authors [25] reported the phylogenetic analysis of bacteria associated with the four parts of *Laminaria saccharina* (*Saccharina lassitima*) and how these populations differed over two seasons, using DDGE and 16S rRNA gene clone libraries. Bengtsson and co-workers also studied the spatial variations between microbial populations found on the meristem and blade regions of *Laminaria hyperborea* [50]. At the same time, the bacterial populations associated with *Nereocystis luetkeana* and *Macrocystis pyrifera* blade and meristem have also recently been reported [24]. However, no such analysis has to date has been conducted on *Laminaria digitata*. Thus, the work presented here aims to bridge that gap and provide a comprehensive insight into the microbial population profile of the different regions of *Laminaria digitata*, using next generation Illumina sequencing which targets the 16S rRNA V3-V4 gene region.

Although this study lacks seawater and substratum controls, it is nevertheless consistent with current literature on marine microbial ecology. Our results show that *Laminaria digitata* supports a diverse range of epiphytic bacteria from phyla *Proteobacteria*, *Bacteroidetes*, *Planctomycetes*, *Actinobacteria*, *Firmicutes* and *Verrucomicrobia*, all of which are known to associate with macroalgal surfaces [31, 50, 51]. Previous studies also report that some bacterial genera identified here, including *Winogradskyella* [52], *Granulosicoccus* [53] and *Aquimarina* [54], possess features such as alginate degradation which would contribute to their growth and survival on the surface of alginate-rich brown algae such as *Laminaria digitata*. Furthermore, although equal volumes of nucleic acids were pooled from three separate individuals (*n* = 3 individuals per algal region per sampling time) and triplicate PCRs, these were not precisely equimolar. While this may have minor effects on the relative abundances reported in this study, it is still evident that differences exist between the components of the bacterial community found in the different algal regions, for example, the bacterial genus *Streptomyces* was not identified in the holdfast region but was present in other thallus regions (Fig 3).

The PCoA plot (Fig 5) and heatmap (Fig 7) further reveal that *L*. *digitata*-associated bacterial population cluster according to the different regions. Some variants within the same bacterial genus, for example *Blastopirellula_0007*, also displayed preferential abundance in specific algal regions. Such observations, in addition to our statistical analysis, suggest that spatial variation has a significant effect on the microbiome of *L*. *digitata* in this study. Similar patterns of spatial variation has also previously been described in different parts of an individual algal thallus or land plant [24, 55]. The leaf and root tissue of *Arabidopsis thaliana* for example differ in their epiphytic bacterial composition [55] while the associated microbiota of the meristem of the kelp *Nereocystis luetkeana* has a greater OTU richness than the apical blade tissue. Functional and nutritional requirements within the different structural parts of an individual algal thallus are unique, thus creating a distinct morphological niche for each part. Stipes in *Fucus vesiculosus*, for example, have the lowest nitrogen assimilation rate, whereas *Laminaria* meristems are rich in nitrogen [56]. It might therefore be expected that the microbial populations associated with the different thallus regions would differ accordingly, as we observed here with *L*. *digitata* in this study, with significant differences seen between the bacterial community profiles associated with the holdfast, stipe, meristem and blade regions of this brown seaweed (Fig 5).

Interestingly, in our study, the holdfast was identified as the most diverse region in *L*. *digitata*, with remarkably higher levels of *Aquimarina*, *Roseobacter* and *Maribacter*, as well as unclassified ASVs, when compared to other regions, while the meristem region was the least diverse and was characterized by a strong presence of *Blastopirellula*, *Litorimonas* and *Granulosicoccus* (Fig 3). While this observation differs from that of Staufenberger and co-workers [25] who reported the holdfast as the least diverse tissue in *Laminaria saccharina* (*Saccharina latissima*), regarding its epiphytic bacterial community, it further demonstrates the uniqueness of bacterial communities associated with different seaweeds, including species with close phylogenetic relationships. The holdfast in kelp species is embedded in heterogeneous sediments which are rich in nutrients, due to the extensive settlement of chemicals and exudates from seaweeds and other surrounding marine species [57]. These sediments which consist of organic and inorganic matter with various physical and chemical properties [55] are thus likely to be attractive for colonization by a wide range of diverse bacterial species.

Furthermore, only 32 ASVs (1.1% of 2,908 ASVs) were common to the different algal thallus regions. Tujula and co-workers previously reported that *Ulva australis* sampled across different seasons shared 60% of DDGE bands, and proposed the existence of a core community of bacterial species on algal surfaces [58]. However, taxonomic analysis in this present study demonstrates that the core epiphytic community of *Laminaria digitata* i.e. a cohort of taxa present on all algal regions is not densely populated, but rather, its associated bacterial populations are unique to their morphological region. This observation suggests that a broad range of bacterial groups exits that are capable of performing similar functional roles that are required for the sustenance of the macroalgal ecosystem [17, 59]. This concept of functional redundancy implies that the recruitment of bacterial groups on macroalgae may depend on their functional competency, rather than taxonomy [60], offering the algal-bacterial community structure a degree of resilience to disturbance [59]. However, the lottery hypothesis [17, 60, 61], further suggests that within a group of functionally competent species, the assembly and recruitment of the community can be stochastic [23]. Functional redundancy has been demonstrated in algal-associated bacterial communities and also in soil-derived microbial populations [62, 63]. While the epibacterial communities associated with *Ulva* species from different geographic locations lacked a taxonomical core bacterial community, further genetic analysis revealed a functional core set of genes related to biofilm formation and in genes responding to environmental stimuli [64].

In conclusion, this study has examined the microbial populations associated with the brown alga *Laminaria digitata* [23]. Here, we observed significant differences between the algal thallus regions, specifically the holdfast, stipe, blades, and meristem. Coupled with morphological differences, the functional requirements of each region create distinct biogeographic signatures which drive the composition and structure of the bacterial populations associated with *Laminaria digitata*, and result in specific communities which lack a dense core population.

## Supporting information

S1 Table Sampling metadata of *Laminaria digitata*-associated bacterial communities. (DOCX)

S2 Table Final ASV table. Abundances of amplicon sequence variants (ASVs) of bacterial communities associated with the holdfast (HF), stipe (SP), meristem (MST) and blade (BD) of *Laminaria digitata*.
(XLSX)

S3 Table Taxonomy table. Genus level taxonomical classification of amplicon sequence variants (ASVs) identified in *Laminaria digitata*.
(XLSX)

S4 Table Statistical analysis (Kruskal-Wallis rank sum test and Dunn post-hoc test) of the differences between the epibacterial communities associated with different parts of *Laminaria digitata* including holdfast (HF), stipe (SP), meristem (MST) and blade (BD). (DOCX)
