# Supplementary material for: Diversity of bacteria populations associated with different thallus regions of the brown alga Laminaria digitata
Source: PLoS One. 2020 Nov 25;15(11):e0242675. doi: 10.1371/journal.pone.0242675 (PMC7688147; doi:10.1371/journal.pone.0242675)
Supplement: S1 Table — (DOCX) [file pone.0242675.s001.docx]

**S1 Table. Sampling metadata of *Laminaria digitata*-associated bacterial communities**

| **Sample ID** | **Sampling month** | **Sampling year** | **Algal region** |
| --- | --- | --- | --- |
| HF1 | APRIL | 2016 | HOLDFAST |
| HF2 | JULY | 2016 | HOLDFAST |
| HF3 | NOVEMBER | 2016 | HOLDFAST |
| HF4 | JANUARY | 2017 | HOLDFAST |
| SP1 | APRIL | 2016 | STIPE |
| SP2 | JULY | 2016 | STIPE |
| SP3 | NOVEMBER | 2016 | STIPE |
| SP4 | JANUARY | 2017 | STIPE |
| MST1 | APRIL | 2016 | MERISTEM |
| MST2 | JULY | 2016 | MERISTEM |
| MST3 | NOVEMBER | 2016 | MERISTEM |
| MST4 | JANUARY | 2017 | MERISTEM |
| BD1 | APRIL | 2016 | BLADE |
| BD2 | JULY | 2016 | BLADE |
| BD3 | NOVEMBER | 2016 | BLADE |
| BD4 | JANUARY | 2017 | BLADE |
