# Supplementary material for: Diversity of bacteria populations associated with different thallus regions of the brown alga Laminaria digitata
Source: PLoS One. 2020 Nov 25;15(11):e0242675. doi: 10.1371/journal.pone.0242675 (PMC7688147; doi:10.1371/journal.pone.0242675)
Supplement: S4 Table — (DOCX) [file pone.0242675.s004.docx]

**S4 Table. Statistical analysis (Kruskal-Wallis rank sum test and Dunn post-hoc test) of the differences between the epibacterial communities associated with different parts of *Laminaria digitata* including holdfast (HF), stipes (SP), meristem (MST) and blades (BD).**

Krsukal-Wallis chi-squared = 11.404, df = 3, p value = 0.009729

| **Comparison** | **Z** | **P.unadj** | **P.adj** |
| --- | --- | --- | --- |
| BD - HF | 1.4852213 | 0.137485208 | 0.206227811 |
| BD - MST | -1.7080045 | 0.087635505 | 0.175271011 |
| HF - MST | -3.1932258 | 0.001406929 | 0.008441572 |
| BD - SP | -0.9653939 | 0.334347578 | 0.401217094 |
| HF - SP | -2.4506152 | 0.014261233 | 0.042783700 |
| MST - SP | 0.7426107 | 0.457717434 | 0.457717434 |
